# The discovery of the mammalian fusome

**DOI:** 10.7554/eLife.110713

**Published:** 2026-02-18

**Authors:** Michael Buszczak, Anirban Dasgupta

**Affiliations:** 1 Department of Molecular Biology, UT Southwestern Medical Center Dallas United States

**Keywords:** oocyte development, germline cyst, fusome, apical-basal polarity, cell cycle, Mouse

## Abstract

The presence of an organelle called the fusome in species as diverse as *Drosophila* and mammals indicates an ancient, conserved programme of germ cell development.

**Related research article** Pathak M, Spradling AC. 2025. Mouse germline cysts contain a fusome that mediates oocyte development. *eLife* **14**:RP109358. doi: 10.7554/eLife.109358.

Germ cells – the cells that make eggs and sperm – are cellular time travelers, linking together the past, present, and future generations of sexually reproducing species. Germ cells must preserve their genetic and cytoplasmic integrity to make functional eggs and sperm, which requires unique developmental strategies (Cinalli et al., 2008). In wide range of species, female germ cells divide to form germline cysts in which the cells are connected by structures called ring canals. Previous studies suggest this arrangement helps to coordinate decisions about the fate of the germ cells, cytoplasmic transport, and various other processes (de Cuevas et al., 1997).

Only a small subset of the cells within a germline cyst matures into oocytes, which give rise to fertilizable eggs. The remaining ‘nurse’ cells provide cytoplasm, organelles, and molecular components that are essential for oocyte growth. First described more than a century ago in histological studies of insects, a specialized organelle called the fusome extends through the ring canals (Lin et al., 1994). The fusome plays a pivotal role in determining whether a cell becomes an oocyte by organizing the microtubule network within the cyst (Grieder et al., 2000), maintaining cell-cell interconnections (Snapp et al., 2004), and ensuring the polarized transport of various organelles and cytoplasmic components (de Cuevas and Spradling, 1998).

Fusome-like structures have been identified in diverse organisms, including *Hydra*, *C. elegans*, *Drosophila*, and *Xenopus*, indicating deep evolutionary conservation. Among these, the composition and function of the fusome has been studied in most detail in *Drosophila*, through a combination of fluorescent immunostaining, confocal microscopy, and live-cell imaging (de Cuevas and Spradling, 1998; Grieder et al., 2000; Lighthouse et al., 2008; Lin et al., 1994; Snapp et al., 2004). In *Drosophila*, the fusome has a branched structure and is composed of endoplasmic reticulum-derived vesicles and a dense meshwork of proteins that are crucial for oocyte specification (Figure 1A). As the cyst grows, the fusome branches and becomes asymmetrically distributed, marking the future oocyte and directing the polarized transport of organelles and cytoplasmic components.

**Figure 1.**
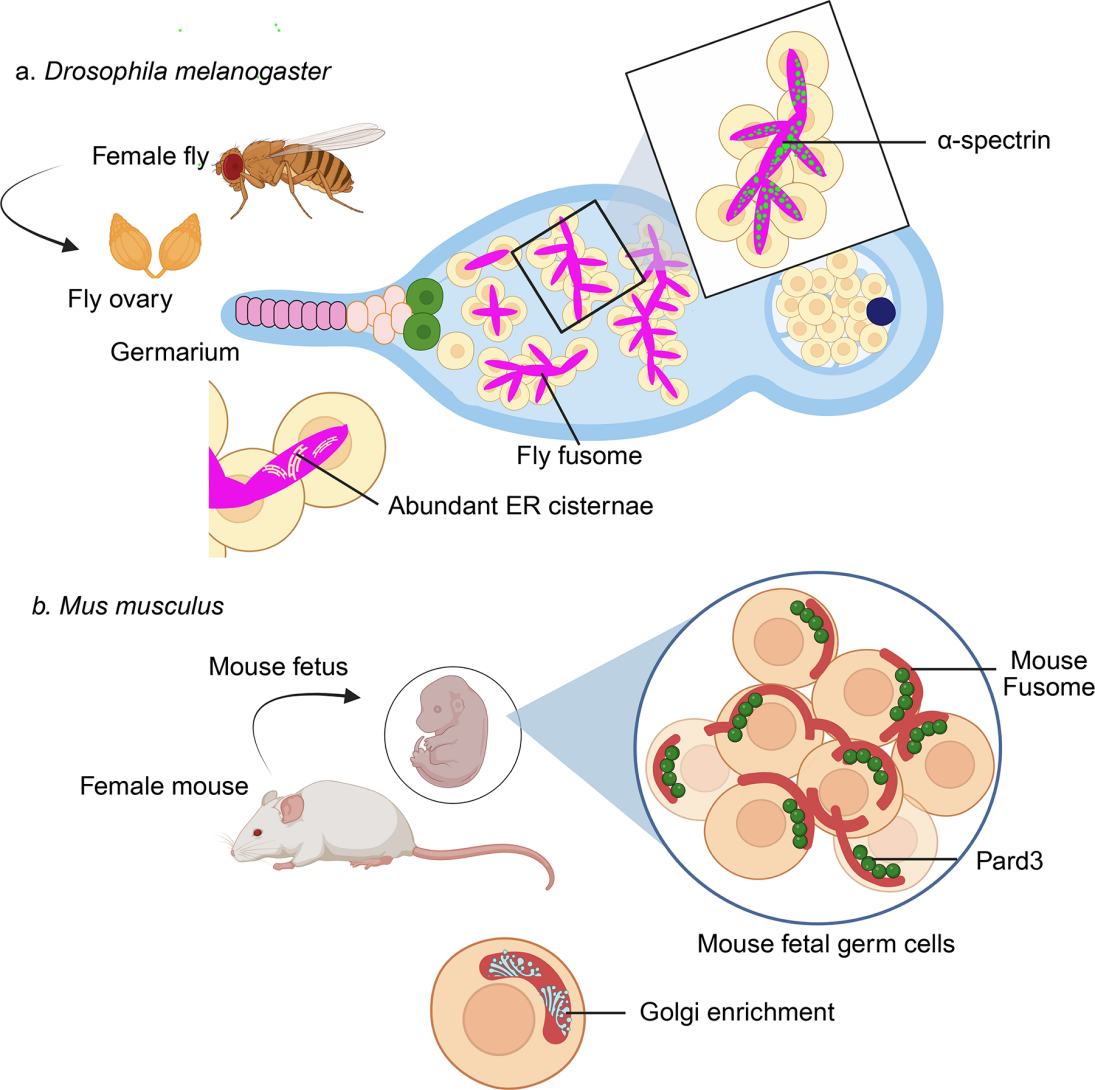
Comparing the fusome in *Drosophila* and mice. (**A**) In *Drosophila*, oocytes are formed in a part of the ovary called the germarium. This region contains cells called terminal filaments (dark pink), cap cells (pale pink), germline stem cells (green), cystoblast cells (beige), and germline cysts that can contain two, four, eight or sixteen cells. The cysts with four or more cells also contain a thick, branched organelle called the fusome (purple). In *Drosophila* the fusome contains alpha-spectrin (lime green) and other spectrin proteins (top right), and large numbers of ER cisternae (orange; bottom left) that connect the cells in the cyst. On the right, fifteen nurse cells (beige) support the development of the oocyte (dark blue). (**B**) Within mouse germ cells (beige), fusomes (brick red) are branched and asymmetric. In contrast to *Drosophila*, the mouse fusome is highly enriched with Golgi membranes (cyan; bottom left), which is consistent with high levels of protein secretion during the development of germ cell cysts in mammals. Pard3 (dark green), a protein involved in asymmetric cell division and polarized growth, also localizes around the asymmetric fusomes. ER: endoplasmic reticulum. Created with BioRender.com.

Previous work has shown that female mice produce germline cysts (Pepling and Spradling, 2001), but it has not been clear if mammalian germ cells also possess a fusome-like structure. Now, in eLife, Madhulika Pathak and Allan Spradling at the Carnegie Institute for Science report compelling evidence for the presence of fusomes in mouse germline cysts (Pathak and Spradling, 2025). These fusomes are enriched in Golgi membranes, endoplasmic reticulum, endosomal vesicles, and microtubules (Figure 1B). Pathak and Spradling show that the mouse fusome is a dynamic structure that forms early during cyst development, and often becomes asymmetrically enriched in future oocytes. Moreover, mouse fusomes associate with Pard3, a protein involved in asymmetric cell division and polarized growth, which is consistent with the idea that fusomes help to direct cyst polarity across different species of animals.

Despite these similarities, there are important differences between the mouse fusome and the *Drosophila* fusome. The latter contains a robust spectrin-based cytoskeletal scaffold that confers mechanical stability and structural rigidity, while alpha-spectrin helps to regulate the division and differentiation of cyst cells (de Cuevas et al., 1996).

By contrast, the mouse fusome lacks alpha-spectrin, and instead shows enhanced association with Golgi and endosomal trafficking machinery. This divergence likely reflects species-specific demands for protein processing, membrane transport, and metabolic regulation during oocyte development.

Strikingly, the mouse fusome likely has important roles in controlling the quality of organelles, proteins and other macromolecules in the cyst. Fusome-associated compartments are enriched in proteins involved in the unfolded protein response, lysosomal markers, and proteasome-related proteins. This suggests that the mouse fusome may serve as a hub for selective protein degradation and lysosome-dependent turnover of remnant nurse cells. By preferentially directing high-quality organelles and macromolecules toward the oocyte, the fusome ensures that the next generation begins life with rejuvenated cytoplasmic components. These new discoveries underscore the remarkable conservation of germline biology across 500 million years of evolution.

The identification of a functional fusome in mice raises important questions. For example, the mechanisms by which fusomes contribute towards germ cell rejuvenation remains poorly understood. Future efforts may reveal how fusome-mediated transport intersects and coordinates pathways involved in mitochondrial quality control, proteostasis, and metabolic remodeling. Furthermore, exploring the extent to which germline cysts in other mammals, including humans, have comparable fusome-like structures – and the possibility that their dysfunction contributes to infertility or age-related declines in oocyte quality – remains important work for the future. A better understanding of mammalian fusome biology could have significant clinical implications in reproductive medicine, and inspire novel strategies to preserve fertility, mitigate reproductive aging, and improve oocyte quality for assisted reproductive technologies, such as in vitro fertilization.
